# Neuroprotection and Neuroregeneration in Alzheimer's Disease

**DOI:** 10.1155/2012/864138

**Published:** 2012-06-11

**Authors:** Kiminobu Sugaya, Moussa B. H. Youdim, Agneta Nordberg, K. S. Jagannatha Rao

**Affiliations:** ^1^College of Medicine, University of Central Florida, Orlando, FL 32827, USA; ^2^Eve Topf and US National Parkinson Foundation, Centers of Excellence for Neurodegenerative Diseases Research and Teaching, Technion-Rappaport Faculty of Medicine, Haifa, Israel; ^3^Department of Biology, Yonsei University, Seoul, Republic of Korea; ^4^Karolinska Institutet, Alzheimer Neurobiology Center, Karolinska University Hospital Huddinge, Stockholm, Sweden; ^5^Institute for Scientific Research and Technology Services (INDICASAT), Clayton, City of Knowledge, Panama

Neurodegeneration in Alzheimer's disease (AD) is thought to be initiated by a cascade of neurotoxic events that include oxidative stress, brain iron dysregulation, glutamate excitotoxicity, nitric oxide, inflammatory process, neurotoxic processing resulting from misfolding, and aggregation of Abeta peptide, as a possible consequence of the demise of ubiquitin-proteasome system (UPS) which is demonstrated neurochemically and by transcriptomics and proteomic profiling. AD is benefitted from the symptomatic effects of cholinesterase inhibitors and glutamate antagonist (memantine), which act on a single molecular target. Such drugs have limited symptomatic activities, and current pharmacological approaches have severe limitations in their ability to be neuroprotective and to modify the course of the disease, offering incomplete and transient benefit to patients. Yet in laboratory and animal models, a number of drugs have demonstrated the ability to be neuroprotective, but in clinical trials, they have failed as a form of symptomatic treatment and disease modification. This situation is not different from that of Parkinson's disease or amyotrophic lateral sclerosis, where the same problems exist. There are a number of valid reasons why we have failed to alter the course of these progressive neurodegenerative disorders. First and foremost, the models employed *in vitro* and *in vivo* are not true representations of complex disease as seen in man. Most of the effort has been in the direction of preventing the formation and overexpression of Abeta peptide in transgenic mice expressing Abeta peptide and plaques. Yet in these animals, there is no process of neurodegeneration. Yet one must question whether the disease is a disorder of Abeta-peptide-induced plaque formation resulting in the cognitive decline or if other processes are involved. The hope is that the newly developed rat transgenic model, which emulates many features of AD, will advance the pathological understanding of the disease and may lead to the development of new therapeutic strategies. The complex pathology of AD pathways includes changes in gene expression, protein metabolisms, response of receptors, level of neurotransmitters, activity of kinase, and signaling pathways. The most important events in neuroprotection and neuroregeneration are the selection of drugs that include synthetic products, natural products, amyloid synthesis, hormonal balance, and nanoparticles intended for a variety of biochemical targets such as oxidative stress. This special issue provides a new knowledge based on therapeutic candidates designed to act on multiple neural and biochemical targets involved in the neurodegenerative process and to possess neuroprotective and neurorestorative activities.

*Kiminobu Sugaya* *Kiminobu Sugaya* *Moussa B. H. Youdim* *Moussa B. H. Youdim* *Agneta Nordberg* *Agneta Nordberg* *K. S. Jagannatha Rao* *K. S. Jagannatha Rao*

